# Effects of yoga in men with prostate cancer on quality of life and immune response: a pilot randomized controlled trial

**DOI:** 10.1038/s41391-021-00470-w

**Published:** 2021-11-23

**Authors:** Dharam Kaushik, Pankil K. Shah, Neelam Mukherjee, Niannian Ji, Furkan Dursun, Addanki P. Kumar, Ian M. Thompson, Ahmed M. Mansour, Richapriya Jha, Xiaoyu Yang, Hanzhang Wang, Nydia Darby, J. Ricardo Rivero, Robert S. Svatek, Michael A. Liss

**Affiliations:** 1 Department of Urology, University of Texas Health San Antonio, San Antonio, TX USA; 2 Mays Cancer Center, University of Texas Health San Antonio, MD Anderson Cancer Center, San Antonio, TX USA; 3 Department of Molecular Medicine, University of Texas Health San Antonio, San Antonio, TX USA; 4 Christus Santa Rosa Health System, San Antonio, TX USA; 5 Nydia Yoga Therapy Studio, San Antonio, TX USA

**Keywords:** Cancer therapy, Cancer therapy

## Abstract

**Background:**

Diagnosis and treatment of prostate cancer is associated with anxiety, fear, and depression in up to one-third of men. Yoga improves health-related quality of life (QoL) in patients with several types of cancer, but evidence of its efficacy in enhancing QoL is lacking in prostate cancer.

**Methods:**

In this randomized controlled study, 29 men newly diagnosed with localized prostate cancer were randomized to yoga for 6 weeks (*n* = 14) or standard-of-care (*n* = 15) before radical prostatectomy. The primary outcome was self-reported QoL, assessed by the Expanded Prostate Index Composite (EPIC), Functional Assessment of Cancer Therapy-Prostate (FACT-P), Functional Assessment of Chronic Illness Therapy-Fatigue (FACIT–F), Functional Assessment of Cancer Therapy-General (FACT-G) at baseline, preoperatively, and 6 weeks postoperatively. Secondary outcomes were changes in immune cell status and cytokine levels with yoga.

**Results:**

The greatest benefit of yoga on QoL was seen in EPIC-sexual (mean difference, 8.5 points), FACIT-F (6.3 points), FACT-Functional wellbeing (8.6 points), FACT-physical wellbeing (5.5 points), and FACT-Social wellbeing (14.6 points). The yoga group showed increased numbers of circulating CD4+ and CD8+ T-cells, more production of interferon-gamma by natural killer cells, and increased Fc receptor III expression in natural killer cells. The yoga group also showed decreased numbers of regulatory T-cells, myeloid-derived suppressor cells, indicating antitumor activity, and reduction in inflammatory cytokine levels (granulocyte colony-stimulating factor [0.55 (0.05–1.05), *p* = 0.03], monocyte chemoattractant protein [0.22 (0.01–0.43), *p* = 0.04], and FMS-like tyrosine kinase-3 ligand [0.91 (−0.01, 1.82), *p* = 0.053].

**Conclusions:**

Perioperative yoga exercise improved QoL, promoted an immune response, and attenuated inflammation in men with prostate cancer. Yoga is feasible in this setting and has benefits that require further investigation.

**Trial registration:**

clinicaltrials.org (NCT02620033).

## Introduction

In 2020, there were 191,930 new prostate cancer cases in the US [1]. A diagnosis of prostate cancer may have profound psychological effects that contribute to poor physical, emotional, and social quality of life (QoL). Up to 30% of men diagnosed with prostate cancer experience significant anxiety, fear, and distress concerning the disease and treatment-related complications [2]. Moreover, the risk of suicide is doubled in the year following a diagnosis of prostate cancer [3]. Increasing use of natural approaches such as acupuncture, herbal supplements and vitamins, yoga, meditation, massage therapy and aromatherapy, coupled with the unmet need for effective management of QoL-related symptoms, has created a demand for integrative medicine in these men [4–6].

Many studies have demonstrated that yoga improves health-related QoL and emotional, physical, and mental wellbeing in patients with cancer. Mindfulness defined as “paying attention in a particular way, on purpose, in the present moment, and non-judgmentally”—has been shown to be improved with yoga practice with focus on breath work [7, 8]. Moreover, in addition to improving fitness, flexibility, and muscle tone, yoga lessens anxiety and stress [9–16].

There is also evidence indicating that yoga attenuates oxidative stress and chronic inflammation associated with stressful situations [11, 13, 17]. However, our understanding of the molecular mechanisms involved in these effects remains limited. Furthermore, most of the relevant studies have been performed in breast cancer, and there are limited data in prostate cancer. The aims of this pilot study were to assess the effects of a perioperative yoga exercise program on QoL, fatigue, sexual and urinary function, and mindfulness and on the cellular immune response and proinflammatory marker levels.

## Materials and methods

### Study design and participants

This block randomized, open-label, parallel-group clinical trial included 29 men with prostate cancer who were scheduled for radical prostatectomy. This was a pilot study of a yoga program in men with prostate cancer to elicit a hypothesis and estimate an appropriate effect size. Patients were accrued from September 25, 2015, to February 6, 2019. The study inclusion criteria were as follows: age 30–80 years; pathologically and/or radiographically confirmed new diagnosis of localized prostate cancer; scheduled for radical prostatectomy (robotic-assisted or open); no active synchronous malignancy; not currently practicing yoga and/or meditation; adequate pain control; no neurological or musculoskeletal comorbidity that would interfere with exercise; willingness to be randomized to either study group and undergo phlebotomy; and ability to provide informed consent. Patients with an absolute contraindication to exercise testing or a psychotic, addiction-related, or major cognitive disorder were excluded. The patients were randomized into a yoga group (*n* = 14) that participated in a yoga program for 6 weeks preoperatively and postoperatively and a control group (*n* = 15) that received standard-of-care only. Control group were patients with new diagnosis of prostate cancer who did not undergo yoga intervention prior to their surgery. All patients completed health-related QoL surveys at baseline (6 weeks preoperatively), immediately before surgery, and 6 weeks postoperatively. Blood samples were collected at these three time points for examination of immune cell status and cytokine levels. The full study protocol is provided as Supplementary Text 1.

The study was approved by the institutional review board of Long School of Medicine, UT Health San Antonio (approval: HSC20150406H). Written informed consent was obtained from all study participants.

### Yoga program

The yoga intervention was developed for patients with prostate cancer as a collaborative effort between the Thrivewell Cancer Foundation, a local yoga studio, and the lead author (DK). The program consisted of 60 min of yoga exercise twice weekly for 6 weeks preoperatively (depending on surgeon and theater availability) and for 6 weeks starting 3–6 weeks postoperatively. The yoga sessions were led by certified instructors from the ThriveWell Cancer Foundation and the local yoga studio. Sessions were held at various locations in San Antonio, TX, and participants could choose their most convenient location. We utilized Hatha yoga method—Hatha yoga generally refers to the practice’s focus on use of physical postures. Hatha yoga was combined with focused attention on gentle breath while moving with awareness through the practice to gently mobilize major joint in the body. The study practice also provided the breathing and pelvic floor engagement awareness in seated meditation at the beginning of yoga practice. Each participant was shown how to perform yoga correctly and safely, with tailoring of exercises to their comfort level. The instructors monitored patients’ progress by observing their ability to breathe smoothly, rhythmically, and continuously while performing yoga. The study was performed in San Antonio, Texas.

### Clinical outcomes

Health-related QoL was assessed using the Functional Assessment of Cancer Therapy-Prostate (FACT-P) scale. FACT-P is a modification of the FACT scale, a 27-item measure of QoL across the domains of physical, social/family, emotional, and functional wellbeing, and contains 12 additional items specific to the impact of prostate cancer symptoms. Therefore, the FACT-P yields both a prostate cancer-related QoL score and a total QoL score [18]. The Five Facets of Mindfulness Questionnaire was used to evaluate the effects of yoga on everyday mindfulness. This 39-item measure includes five domains (Observe, Describe, Act with Awareness, Non-judging of Inner Experience, and Nonreactivity to Inner Experience) and has been validated in both English and Spanish [19].

Cancer-specific fatigue was measured using the 13-item Functional Assessment of Chronic Illness Therapy-Fatigue (FACIT-F) questionnaire [20], urinary continence using the 7-item Expanded Prostate Index Composite (EPIC) urinary questionnaire [21], and erectile function using the 9-item EPIC-sexual function questionnaire [22]. All three questionnaires have been confirmed to have good reliability and validity.

### Analysis of immune cells

Cryopreserved human peripheral blood mononuclear cells (PBMCs) were obtained at all assessment times and processed for immune analysis as previously described [23].

Briefly, PBMC cryovials were thawed rapidly at 37 °C and washed in 9 ml of warm serum-free RPMI-1640 medium (Corning, Corning, New York, NY). Single-cell PBMC suspensions were quantified for total number of live cells using an automated cell counter (Vi-Cell XR, Beckman Coulter, Brea, CA). After centrifugation at 4 °C and 1200 rpm for 5 min, the cell pellets were resuspended with ice-cold flow buffer (sterile 2% fetal bovine serum in phosphate-buffered saline) at a maximum of 1 × 10^6^ cells with a staining volume of 100 µl on 96-well U-bottom plates; they were then incubated with human Fc blocker for 20 min and stained using fixable viability dye and fluorochrome-conjugated anti-human monoclonal antibodies for 45 min at 4 °C in the dark (Supplementary Table 1). For examination of the cytokine response in immune cells, the thawed PBMCs were resuspended in complete RPMI-1640 medium containing 10% fetal bovine serum, penicillin/streptomycin, and L-glutamine (Corning) on a 96-well U-bottom plate and incubated in a resting state at 37 °C overnight. Next, the cells were stimulated for 5 h using Cell Activation Cocktail (BioLegend, San Diego, CA) at a dilution of 1:500. Cell surface and intracellular staining was then performed using a Fixation/Permeabilization Solution Kit (Cytofix/Cytoperm, BD Biosciences, San Jose, CA). The stained samples were analyzed using an LSRII cytometer (BD Biosciences) with FACS Diva software. Using the gating strategy for immune cell subsets, all live PBMCs were first gated from singlets and fixable viability dye-negative populations. CD4^+^ and CD8^+^ T-cells were gated under a live CD3^+^ population. Regulatory T-cells were gated as live CD3^+^CD4^+^CD25^+^Foxp3^+^ cells and γδ T-cells were gated as live CD3^+^CD4^−^CD8^−^ cells. Natural killer (NK) cells were gated as a live CD3^−^CD56^+^ population and subdivided further into CD56^bright^ and CD56^dim^ NK cells. Myeloid populations were identified as live CD11b^+^ cells. Myeloid cells were further gated into subpopulations as follows: macrophages as live CD11b^+^CD68^+^ cells were divided further into M1 macrophages (live CD11b^+^CD68^+^CD64^+^CD80^+^ cells) and M2 macrophages (live CD11b^+^CD68^+^CD23^+^CD163^+^ cells), MDSCs as live CD11b^+^CD33^+^ cells further subgated into M-MDSCs (live CD11b^+^CD33^+^ HLADR^−^CD15^−^CD14^+^ cells) and G-MDSCs (live CD11b^+^CD33^+^CD15^+^CD14^−^ cells), neutrophils as live CD11b^+^CD15^+^CD14^−^ cells, and dendritic cells as live CD11b^+^CD14^+^CD15^−^ cells. The gating strategy for expression of subsequent cell surface or intracellular markers on each immune cell subset was based on a fluorescence minus one control for each individual marker/monoclonal antibody.

### Evaluation of cytokines

Plasma samples from the 15 patients in the control group and 14 patients in the yoga group were analyzed at the Bioanalytics and Single-Cell Core Laboratory in the Department of Molecular Medicine at UT Health San Antonio. The samples were centrifuged at 10,000 *g* and 4 °C for 10 min. Next, 25 μl of the clarified top portion were collected and analyzed in duplicate using the FlexMap 3D platform system (Luminex, Austin, TX) with the Milliplex MAP human cytokine/chemokine Magnetic Bead Panel-Immunology Multiplex Assay kit (Cat # HCYTMAG-60K-PX38; EMD Millipore, Billerica, MA). The following cytokines and chemokines were analyzed: epidermal growth factor, fibroblast growth factor-2, FMS-like tyrosine kinase (Flt)−3 ligand, fractalkine, granulocyte colony-stimulating factor (G-CSF), granulocyte-macrophage colony-stimulating factor, growth-regulated protein, interferon (IFN)-α2, IFN-γ, interleukin (IL)-1α, IL-1β, IL-1ra, IL-2, IL-3, IL-4, IL-5, IL-6, IL-7, IL-8, IL-9, IL-10, IL-12 (p40), IL-12 (p70), IL-13, IL-15, IL-17A, IFN-gamma-induced protein (IP)-10, monocyte chemoattractant protein (MCP)-1, MCP-3, macrophage-derived chemokine (CCL22), macrophage inflammatory protein-1α/1β, transforming growth factor-α, tumor necrosis factor (TNF)-α, TNF-β, vascular endothelial growth factor, eotaxin/CCL11, and sCD40L.

### Statistical analysis

Domain and subdomain scores for all patient-reported outcomes were calculated as per their respective guidelines and then scaled to a 0–100 score. A higher score indicates better health-related QoL. All continuous variables were evaluated for normality using the Kolmogorov–Smirnov test. Between-group differences in sociodemographic, clinical, and immune parameters were evaluated at baseline using the Student’s *t*-test for continuous variables and the chi-squared test for categorical variables (Fisher’s exact test was used as appropriate). The effect of the intervention was evaluated by calculating the improvement in patient-reported outcome scores, cytokine expression, and numbers of immune cells between baseline and immediately before surgery. Between-group differences were evaluated using the Student’s *t*-test if the variable was normally distributed and the Wilcoxon rank-sum test if not. The significance level was set to α = 0.05 (two-tailed) but not adjusted for multiple comparisons using methods such as Bonferroni correction because we were not testing any a priori hypothesis. The primary outcome was self-reported QoL at baseline, preoperatively, and 6 weeks postoperatively. Secondary outcomes were changes in immune cell status and cytokine levels with yoga. The analysis was restricted to only the first two time points because only two of the 15 participants in the yoga group completed the third time point (per-protocol analysis). All statistical analyses were performed using R software (R Foundation for Statistical Computing, Vienna, Austria).

## Results

**Fig. 1:**
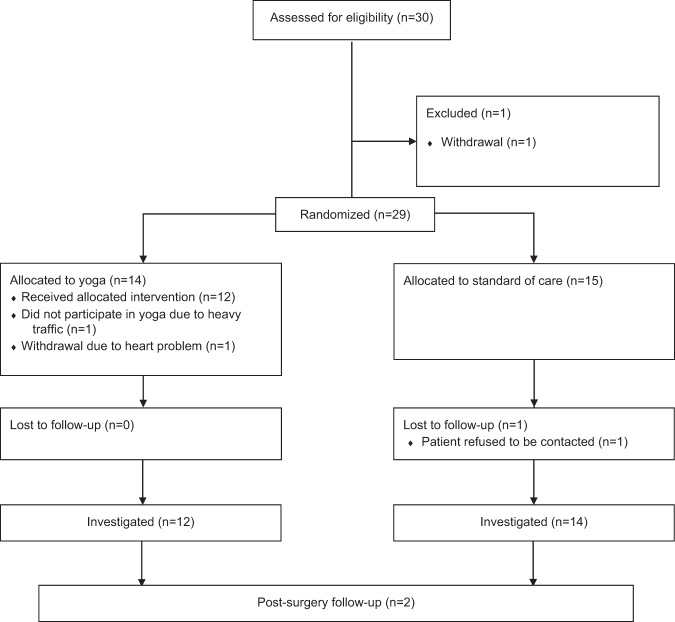
CONSORT diagram. Flowchart of patient enrollment process for the study.

Twenty-nine of 30 patients assessed for eligibility were randomized to the yoga group (*n* = 14) or the control group (*n* = 15). Twelve patients in the yoga group completed their yoga program and could be followed up before surgery and one in the control group was lost to follow-up. Therefore, complete data for 26 patients were available for the analyses (Fig. 1).

### Patient demographics and clinical characteristics

The baseline sociodemographic and clinical characteristics are shown in Supplementary Table 2. The median patient age was 60 years (IQR 59–61) in the control group and 56 years (IQR 55–60.5) in the yoga group. Most of the patients had organ-confined disease. Approximately 23% of the cohort was Hispanic. There was no significant between-group difference in QoL at baseline (Supplementary Table 3).

### Clinical outcomes

**Fig. 2:**
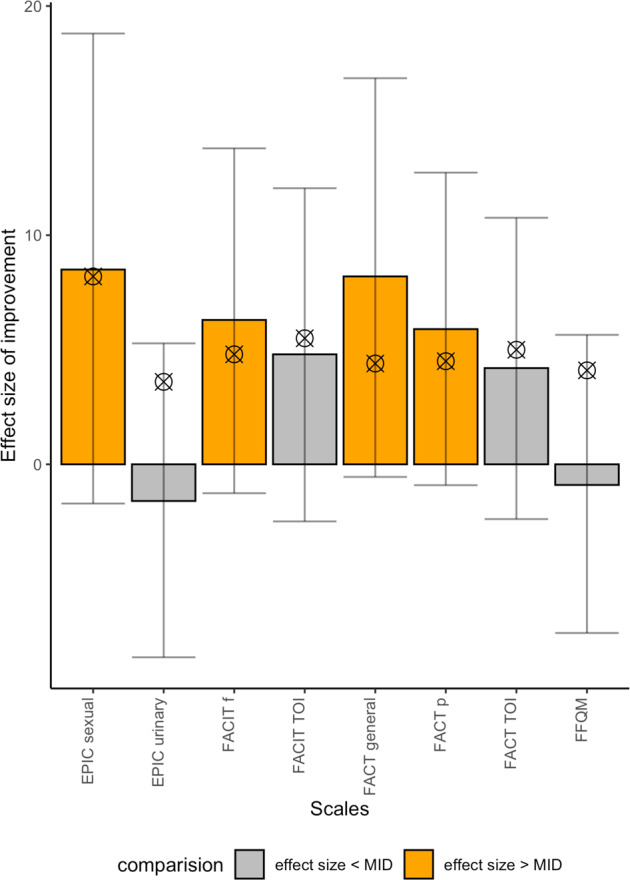
Effect size of the yoga intervention compared to the minimally important difference (MID) with 95% confidence interval. The effect size was calculated as the difference between the postintervention score minus the preintervention score in the yoga group and the postintervention score minus the preintervention score in the control group. The MID was calculated as one-third of the overall standard deviation of the patient-reported scale item at baseline. The ⊗ mark on the graph indicates the MID for the scale item. EPIC Expanded Prostate Index Composite, FACIT Functional Assessment of Chronic Illness Therapy, FACIT-F FACIT-Fatigue, FACIT-G FACIT-General, FACT Functional Assessment of Chronic Illness Therapy, FFOM Five Facets of Mindfulness, TOI Trial Outcome Index.

Table 1 shows the changes in mean scores in both study groups at the scale and subscale levels. Overall, there was a statistically nonsignificant trend towards improvement in sexual function (EPIC questionnaire, yoga vs control: 9.1 vs. 0.6; *p* = 0.098), fatigue (FACIT-F questionnaire, 1.8 vs. −4.5; *p* = 0.098), general QoL (FACT-P: 1.9 vs. −6.3; *p* = 0.065), and prostate-specific QoL (0.6 vs. −5.3; *p* = 0.08). We then calculated the minimally important difference (MID), namely, the minimal effect that would be meaningful to patients, for each patient-reported QoL outcome by stratifying our data to one-third of a standard deviation. This amount of change in the standard deviation has been shown to have a clinically meaningful impact on a patient’s QoL [24, 25]. We then found that the FACT-P, FACT-General, FACIT-F, and EPIC-Sexual scores were improved meaningfully by yoga (Fig. 2). On further substratification of these scales using the MID, there were improvements in the FACT-P physical, social, and functional wellbeing scores and in EPIC-Sexual function (Supplementary Fig. 1) in the yoga group.

**Table 1:** Improvement in patient-reported outcomes by study group at the scale and subscale levels.

Scale level		Study group				
	Domain	Yoga mean (SE)	Control mean (SE)	*P*-value	Effect size with 95% confidence interval	Greater than MID
Expanded prostate cancer index composite						
	Sexual	9.1 (2.6)	0.6 (4.2)	0.098	8.5 (−1.7, 18.8)	Yes
	Urinary	−0.2 (2.7)	1.4 (1.9)	0.638	−1.6 (−8.4, 5.3)	No
Functional assessment of chronic illness therapy						
	Trial outcome index	1.8 (2.5)	−3.0 (2.5)	0.187	4.8 (−2.5, 12.1)	No
	Fatigue	1.8 (2.3)	−4.5 (2.8)	0.098	6.3 (−1.3, 13.8)	Yes
Functional assessment of cancer therapy						
	Trial outcome index	0 (2.5)	−4.2 (2.0)	0.200	4.2 (−2.4, 10.8)	No
	General	1.9 (2.7)	−6.3 (3.2)	0.065	8.2 (−0.6, 16.9)	Yes
	Prostate	0.6 (2.2)	−5.3 (2.5)	0.086	5.9 (−0.9, 12.7)	Yes
Five facet mindfulness questionnaire		−1.5 (2.8)	−0.6 (1.2)	0.783	−0.9 (−7.4, 5.7)	No

Subscale level		Study group				
Expanded prostate cancer index composite		Yoga mean (SE)	Control mean (SE)	*P*-value	Effect size with 95% confidence interval	Greater than MID
	Sexual bother	11.5 (4.7)	3.8 (7.0)	0.376	7.6 (−9.9, 25.1)	No
	Sexual function	8.1 (2.7)	−0.9 (3.2)	0.043	9.0 (0.3, 17.6)	Yes
	Urinary bother	−0.9 (3.3)	1.1 (2.5)	0.635	−2.0 (−10.6, 6.6)	No
	Urinary function	−0.7 (3.5)	1.8 (2.6)	0.577	−2.5 (−11.5, 6.6)	No
	Urinary incontinence	−1.9 (4.5)	−2.6 (2.3)	0.894	0.7 (−10, 11.4)	No
	Urinary obstructive	0.1 (3.0)	4.1 (2.4)	0.308	−4.0 (−12, 4)	No
Functional assessment of cancer therapy						
	Emotional wellbeing	−1.0 (4.3)	−4.2 (3.4)	0.573	3.1 (−8.2, 14.5)	No
	Fatigue	1.6 (2.4)	−0.7 (2.8)	0.536	2.3 (−5.3, 9.9)	No
	Functional wellbeing	1.2 (4.4)	−7.4 (2.8)	0.113	8.6 (−2.2, 19.5)	Yes
	Prostate cancer subscale	−2.3 (2.7)	−3 (2.2)	0.834	0.7 (−6.4, 7.9)	No
	Physical wellbeing	2.7 (3.2)	−2.8 (3.2)	0.241	5.5 (−4, 15)	Yes
	Social wellbeing	4.2 (3.0)	−10.4 (7.9)	0.103	14.6 (−3.3, 32.5)	Yes
Five facet mindfulness questionnaire						
	Awareness	−2.6 (4.2)	1.7 (3.3)	0.429	−4.3 (−15.3, 6.8)	No
	Describing	−1.8 (3.9)	−4.1 (2.4)	0.629	2.3 (−7.4, 11.9)	No
	Non-judging	−8.9 (3.2)	−1.2 (3.1)	0.101	−7.7 (−17, 1.6)	No
	Nonreactive	5.7 (4.3)	3 (2.9)	0.619	2.6 (−8.3, 13.5)	No
	Observing	1.0 (5.8)	−2.2 (4.1)	0.656	3.2 (−11.6, 18)	No

*MID* minimally important difference, *SE* standard error.

### Immune cells

**Fig. 3:**
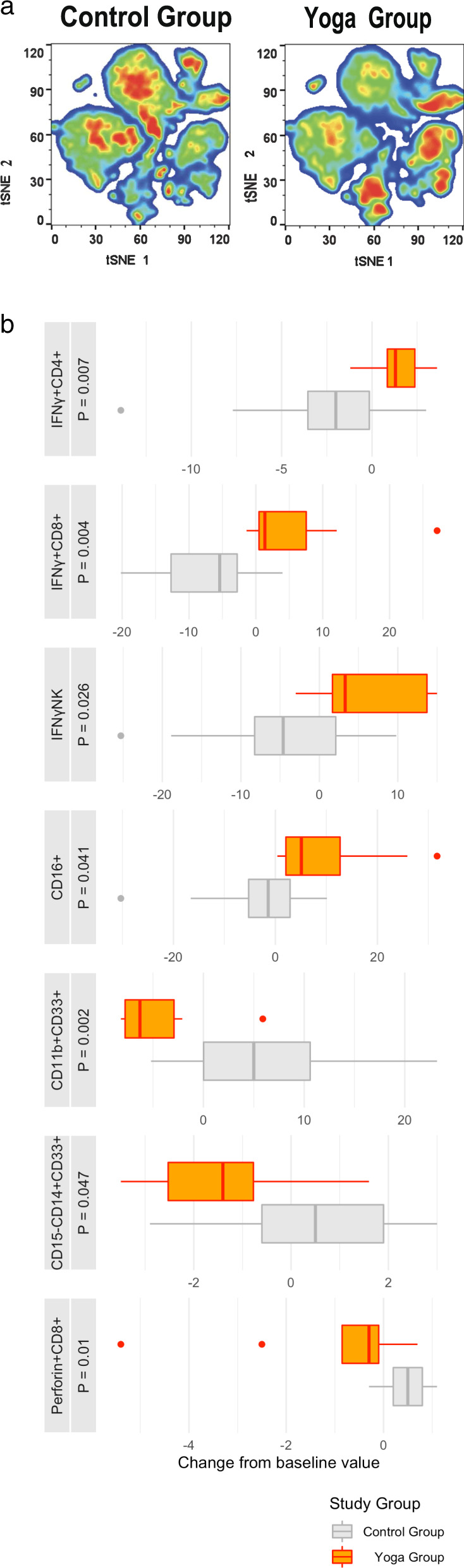
Analysis of immune cell data. **a** A t-distributed stochastic neighbor embedding (tSNE map) showing differences in immune cell phenotypes between the yoga group and the control group. **b** Box and whiskers plot of immune markers (IFNγ+CD4+, IFNγ+CD8+, IFNγNK, CD16+, CD11b+CD33+, CD15-CD14+CD33+, perforin+CD8+) between samples obtained from 13 patients in the control group and those obtained from 6 patients in the yoga group at endpoint.

We characterized lymphocytes from the patients’ blood samples using multi-parametric gating flow cytometry (Supplementary Fig. 2 shows the results and Supplementary Table 1 lists the antibodies used). We then analyzed the immune cell data by creating a t-distributed stochastic neighbor embedding (tSNE) plot, which is a non-linear dimensionality reduction algorithm that provides “big picture” data. A global t-SNE map of PBMCs was obtained using a 12-parameter flow cytometry panel. Using the tSNE algorithm, we identified qualitative phenotypic differences between the yoga group and control group (Fig. 3a). We further delineated the populations of T-cells, NK cells, and subsets of myeloid cells and MDSCs. We then plotted the differences in frequencies and absolute numbers of immune cells between the study groups using box and whisker plots (Supplementary Fig. 3a–e). We identified an increased IFN-γ response in peripheral cytotoxic CD4+ (*p* = 0.007) and CD8+ (*p* = 0.004) cells in the yoga group in comparison with the control group (Fig. 3b). Levels of regulatory T-cells (*p* = 0.029) and MDSCs (*p* = 0.002) were reduced in the yoga group when compared with the control group, indicating enhanced antitumor activity in patients who practiced yoga. We also identified increased Fc receptor III (*p* = 0.041) and IFN-γ (*p* = 0.026) expression in NK cells, indicating a robust immune response as well as antitumor activity.

### Cytokines

**Fig. 4:**
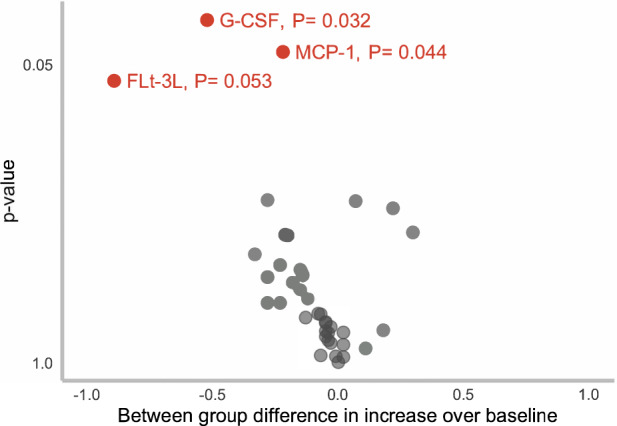
Analysis of cytokine data. Volcano plot showing the difference in increase in expression levels of granulocyte colony-stimulating factor (G-CSF), monocyte chemoattractant protein (MCP-1) and FMS-like tyrosine kinase-3 ligand (Flt-3 ligand) from baseline between the yoga group and the control group granulocyte colony-stimulating factor [0.55 (0.05–1.05), *p* = 0.03], monocyte chemoattractant protein [0.22 (0.01–0.43), *p* = 0.04], and FMS-like tyrosine kinase-3 ligand.

A statistically significant reduction in expression of inflammatory cytokines was identified in the yoga group, namely, G-CSF (0.55 [0.05–1.05]; *p* = 0.032), MCP-1 (0.22 [0.01–0.43]; *p* = 0.044), and Flt-3 ligand (0.91 [−0.01, 1.82]; *p* = 0.053) (Fig. 4). Changes in levels of 38 cytokines from baseline are compared between the study groups in Supplementary Fig. 4 and Supplementary Table 4.

## Discussion

Although there have been some small retrospective studies and one relevant randomized trial [14], the effects of yoga in patients with prostate cancer remain unclear, particularly regarding QoL and its molecular impact. Therefore, we designed this clinical trial to obtain preliminary clinical and translational data.

Our preliminary data add to the literature by providing a molecular explanation for the similar improvements in QoL seen in patients with prostate cancer. The greatest impact of yoga was on sexual function, fatigue, prostate cancer-specific QoL, and physical, social, and functional wellbeing. Our data also indicate that yoga modulates several key immune cells that are important drivers of antitumor activity. Furthermore, the analysis of cytokines/chemokines suggests that yoga attenuates the inflammatory response.

Our data demonstrate a positive effect of yoga in several clinical domains. First, we observed a significant improvement in QoL in the perioperative setting, which was reflected in enhanced physical, social, and functional wellbeing as well as improvement in symptoms of fatigue and stress. These findings are consistent with those of a trial by Ben-Josef et al. [14] in which 50 patients with prostate cancer undergoing radiation therapy were allocated to yoga classes (*n* = 22) or standard-of-care (*n* = 28) for 6–9 weeks. In that study, patients in the yoga group experienced significantly less global fatigue and severity of fatigue than those in the control group (*p* < 0.0001). Moreover, as in our study, sexual health scores were better in their yoga group. Overall, our data and those of Ben-Josef et al. demonstrate that yoga has a positive effect on emotional, physical, and social scores.

Perioperative exercise studies for patients who underwent radical prostatectomy are mostly focused on the urinary continence after the surgery. Few studies with various perioperative exercise interventions studied the impact of exercise on cancer-specific QoL. In their randomized control trial assessing 49 patients, Park et al. [26] reported that a postoperative combined exercise intervention results in improvement of physical function and QoL. In a large systematic review and meta-analyses of 1057 prostate cancer patients enrolled in 13 randomized clinical trials, exercise intervention significantly improved fatigue symptoms [mean difference (MD) 4.83, 95% CI 3.24–6.43; *p* < 0.00001] as assessed according to the Functional Assessment of Cancer Therapy (FACT)-Fatigue scale. Fatigue remained improved at 6 months (MD 3.60, 95% CI 2.80–5.12; *p* < 0.00001). Furthermore, exercise interventions improved QOL measured using the FACT-General (MD 3.93, 95% CI 1.37–5.92; *p* = 0.003) and FACT-Prostate (MD 3.85, 95% CI 1.25–6.46; *p* = 0.04) scales [27].

A recent multi-institutional study examined the effect of yoga on quality of sleep in 410 cancer survivors (>90% female) who were randomly assigned to 4 weeks of yoga (*n* = 206) or standard-of-care (*n* = 204) [9]. Compared with the control group, the yoga group demonstrated significant global improvements in quality, duration, and efficiency of sleep and less use of sleep medication. Longer-term follow-up of that cohort revealed significant improvement in all subdomains of cancer-related fatigue [28].

The Society for Integrative Oncology has produced an evidence-based guideline on use of integrative therapies during and after treatment of breast cancer that has been endorsed by an American Society of Clinical Oncology expert panel [29]. This guideline recommends yoga for reduction of anxiety and stress, amelioration of depression/mood, and improvement of QoL. Our data add to the literature by providing a molecular explanation for the similar improvements in QoL seen in patients with prostate cancer.

There are data suggesting that psychological stress has a negative effect on the adaptive cellular immune response, including decreased production of NK cells and T-cells. Chronic stress results in stimulation of the hypothalamic-pituitary-adrenal axis, which produces glucocorticoids, and the sympathetic-adrenal axis, which produces catecholamines [30]. Leukocytes have receptors for these stress-related hormones and can modulate their binding [31]. T-cells have more of these receptors and are exquisitely sensitive to fluctuations in stress hormone levels. Recent data show that stress and anxiety may lead to metabolic changes that impair the function of CD4+ T-cells [32]. In the present study, we identified a robust IFN-γ response in CD4+ and CD8+ cells, increased expression of the Fc receptor (CD16) in NK cells, and decreases in numbers of regulatory T-cells and MDSCs. These findings, although only hypothesis-generating, point to a strong immune response, less stress, and better QoL in patients with prostate cancer who practice yoga. Future studies are needed to clarify the impact of yoga on T-cell subpopulations.

Research has shown a relationship between persistent fatigue and overactivation of the inflammatory network in patients with cancer. Other studies have shown an association between QoL indicators, including fatigue, anxiety, and depression levels, and increased production of proinflammatory cytokines, including IL-6 and TNF-α [33]. Our data show that 6 weeks of a yoga exercise program reduced fatigue and expression of proinflammatory markers, including G-CSF, MCP-1, and Flt-3 ligand. G-CSF has been shown to activate production of endothelial cells and cytokines and to promote angiogenesis [34]. MCP-1 is a chemokine that is expressed by glial cells and neurons. Higher plasma MCP-1 levels are associated with more rapid and severe cognitive decline secondary to neuronal loss whereas lower levels are neuroprotective [35, 36]. There is emerging data showing that MCP-1 acts as a potent chemotactic factor regulating stromal—epithelial cell in prostate cancer. It regulates prostate cancer motility and proliferation at the site of the bone microenvironment and overexpression of its receptors (CCR2 and CCRL1) may contribute to the progression and biochemical failure of prostate cancer [37]. Finally, higher expression levels of Flt-3 ligand have been linked to an autoimmune response and to chronic inflammatory responses in the lung, central nervous system, and gastrointestinal tract [38]. By lessening inflammation and fatigue and improving mood, yoga is an ideal exercise that can be modified for individuals with a sedentary lifestyle or functional limitations [11, 16, 28, 39–41]. Our pilot data are consistent with those of previous retrospective studies demonstrating the beneficial effects of yoga on several psychological and QoL outcomes, add granularity at the molecular level, and identify putative inflammatory markers for future research.

This study has several limitations. First, the study cohort was small. Second, assessments were performed at only two time points and adherence rates are not available. Third, given that we were not testing any a priori hypothesis, the significance level was not adjusted for multiple comparisons using methods such as Bonferroni correction. Therefore, our data is hypothesis-generating and further research is required.

In conclusion, our present findings indicate that yoga improves QoL, generates a robust immune response, and attenuates expression of key inflammatory cytokines in men with newly diagnosed prostate cancer. Our data shows that patients are motivated to perform yoga in the preoperative setting (no attrition) but not in the postoperative period (high attrition). Larger-scale studies are needed to replicate these results. Future studies should incorporate a translational component to clarify the mechanism via which yoga improves QoL and examine the effects of yoga on progression and recurrence of prostate cancer.

## Supplementary information

Supplementary Material

## Data Availability

The principal investigator (DK) and biostatistician (PS) had full access to all the data in the study and takes responsibility for the integrity of the data and the accuracy of the data analysis.
