# Supplementary material for: Effects of yoga in men with prostate cancer on quality of life and immune response: a pilot randomized controlled trial
Source: Prostate Cancer Prostatic Dis. 2021 Nov 23;25(3):531–8. doi: 10.1038/s41391-021-00470-w (PMC9124736; doi:10.1038/s41391-021-00470-w)
Supplement: Supplementary file 1 — Supplementary Material [file 41391_2021_470_MOESM1_ESM.docx]

**SupplementARY INFORMATION**

**Effects Of Yoga In Men With Prostate Cancer On Quality Of Life And Immune Response: A Pilot Randomized Controlled Trial**

**SUPPLEMENTARY TEXT 1**

**Study methodology**

***Screening visit***

Research staff reviewed the clinic schedules to identify potential study participants. Information regarding study eligibility for each patient was made available to the treating physician who provided clinical care and saw the patient in clinic. The treating physician, a physician assistant, or a nurse practitioner made the initial contact with patients whose pre-screening/chart review indicated that they may be eligible to participate in the study. Patients who were interested in participating in the study were able to contact the research staff directly, and if deemed eligible for participation, were scheduled for a research appointment.

The following inclusion criteria were applied:

- Age 30–80 years
- A pathologically and/or radiographically confirmed diagnosis of localized prostate cancer
- Scheduled to undergo robotic-assisted or open radical prostatectomy
- No other active primary malignancy apart from prostate cancer
- Currently not practicing yoga as a form of exercise and/or meditation
- Effective pain control
- No neurological or musculoskeletal comorbidity that could interfere with exercise
- No known physician-diagnosed contraindications to exercise testing
- Willing to be randomized to either study group
- Willing to participate in yoga sessions for 12 weeks if randomized to the intervention group
- Willing to undergo phlebotomy
- Able and willing to provide informed consent.

Patients diagnosed to have a psychotic illness or major cognitive disorder were excluded, as were those with current addiction-related issues. Screening information collected for ineligible participants was shredded immediately.

Sociodemographic data, including age, marital status, living situation, employment status, occupation, and educational level, and clinical data, including history of treatment for prostate cancer, tumor characteristics, disease stage, and comorbidities, were collected by chart review and interviews.

Patients deemed to meet the pre-screening criteria were first approached in a private examination room by the attending physician, who could also be a research investigator. If the patient expressed interest in participating in the study, he was provided with further information about the research, any questions were answered, and he was invited to attend for a baseline assessment.

***Baseline visit***

Baseline assessments were performed by an IRB-approved member of the research team at the Cancer Therapy Research Center/Medical Arts and Research Center, The University of Texas Health Science Center at San Antonio, or via the South Texas Veterans Health Care System. On arrival, the patient was escorted to a private interview room, where he was given an overview of the study by the principal investigator, a co-investigator, or the study coordinator. The patient was then provided with a copy of the consent form that he could take home to read and discuss with his family, friends, and/or family doctor. If still interested, the potential study participant was scheduled to return for an assessment. At the return visit, the same member of the research team answer any questions/concerns regarding informed consent. The voluntary nature of participation, purpose of the study, study procedures, risks and benefits, collection of protected health information, confidentiality, and participant rights were discussed. If still willing to take part in the study, the patient signed the consent form. A copy of the signed consent form was given to the patient.

At the same visit, two 10-mL blood samples were drawn for research purposes. The patient then completed a series of study questionnaires designed to collect information on demographics, self-reported current physical activity level, and health-related quality of life. Patients were allowed to return the completed questionnaires to the study team by email or post if they could not complete them within the time allotted for the visit. A “health check” was also performed, during which the patient’s height, weight, and vital signs were recorded

Next, a six-minute walk test (6MWT) was performed according to the American Thoracic Society guideline under the supervision of an IRB-approved study team member. The patient was instructed to wear comfortable clothing and shoes, not to exercise vigorously for 2 hours before the test, have a light meal beforehand, and to continue on their usual prescribed medication. The test was performed in a hallway/corridor at the assessment site. The patient was required to sit at rest in a chair for 10 min before starting the test. During this time, the study investigators checked for any contraindications to the 6MWT and measured the patient’s resting pulse rate, blood pressure, and oxygen saturation. Shortness of breath and overall fatigue at baseline were assessed using the Borg Dyspnea Scale. The patient was instructed to walk back and forth as many times as possible in 6 min along a 30-m course marked with two small cones to indicate turnaround points. The patient could rest or stop the test at any time. No running or jogging was permitted. The post-test Borg Dyspnea Scale score, pulse, and oxygen saturation level were remeasured on completion of the 6MWT.

At the end of the baseline visit, each study participant was allocated to a control group or a yoga group using a permuted block randomization procedure with sequentially numbered, opaque, sealed envelopes. Two block sizes (4 and 6) were used to avoid correct anticipation of the randomization sequence. All patients in both study groups underwent standard radical prostatectomy for localized prostate cancer. Patients assigned to the control group were instructed to continue with their normal daily activities and routine before and after their scheduled surgery. Patients assigned to the yoga group attended free supervised yoga sessions for 60 minutes twice per week for 12 weeks.

***Mid-study visit***

The mid-study visit was scheduled to be at the same time as or as close as possible to the patient’s routine preoperative visit. Patients randomized to the yoga group had completed 6 weeks of yoga therapy by this time. After completion of the routine preoperative assessment, two (optional) 10-mL blood samples were again drawn for research purposes. Next, the patient completed the study questionnaires. Height, weight, and vital signs were then recorded. If the patient was unable to attend this visit, the study team offered to send the study questionnaires via email or post according to the patient’s preference and organize follow-up based on the patient’s preferred method of communication. If the completed questionnaires were not received within a week of the mid-study date, a member of the study team contacted the patient by telephone to remind them to do so. In this scenario, the optional research blood samples were not drawn, and the latest height, weight, and vital signs in the medical record were recorded on the patient’s study chart.

***End-of-study visit***

The end-of-study visit was scheduled to take place at least 9 weeks after the date of surgery, preferably at the same time as the routine postoperative visit. During this visit, the patients repeated their 6MWT, completed the end-of-study questionnaires, and provided blood samples for research purposes. Again, each patient had the option of emailing or posting the questionnaires back to the study team if they could not completed them within the time allocated for the study visit. Each patient’s height, weight, and vital signs were again recorded at this visit.

***Withdrawal procedure***

The principal investigator could withdraw a patient from the study at any time in the event of an injury or complication that interfered with the patient’s ability to perform the prescribed yoga exercise. Patients were also able to withdraw from the study at any time, regardless of group allocation. Whenever possible, a patient who was considering withdrawing from the study was encouraged (but not obliged) to attend for a health check, to have blood samples drawn for research purposes, to perform a 6MWT, and to complete the study questionnaires. Reasons for withdrawal were sought at these visits.

**Study calendar**

|  | Baseline visit | Control group performed normal, daily activities  Yoga group participated in a yoga therapy | Mid-study visit | Radical prostatectomy  (standard-of-care) | 3–6 weeks after surgery:  Control group returned to normal daily activities as permitted by the treating physician  Yoga group continued with yoga therapy | End-of-study visit |
| --- | --- | --- | --- | --- | --- | --- |
| Informed consent taken | X |  |  |  |  |  |
| 6MWT | X |  |  |  |  | X |
| Study questionnaires | X*** |  | X*** |  |  | X*** |
| Health check (height weight, vital signs) | X |  | X** |  |  | X |
| Blood samples drawn for research purposes | X |  | X* |  |  | X |

*Optional. **Could be extracted from the data recorded at the most recent visit if the patient could not attend the mid-study visit. ***Could be emailed or sent by post, depending on the patient’s preference

**Assessment of outcomes of interest**

| Outcome | Measures | Method of Assessment |
| --- | --- | --- |
| Demographics |  | Self-reported questionnaire |
| Health history, including height, weight, and vital signs |  | Physical examination, review in EPIC |
| Current physical activity level |  | Self-reported questionnaire |
|  |  |  |
|  |  |  |
| Study processes |  |  |
| Recruitment rate  Adherence  Attrition  Safety |  |  |
|  |  |  |
| Outcomes of interest |  |  |
| Quality of life | FACT-P | Self-reported questionnaire |
| Fatigue | FACT-F | Self-reported questionnaire |
| Recovery of urinary continence | EPIC urinary function | Self-reported questionnaire |
| Recovery of erectile function | EPIC erectile function | Self-reported questionnaire |
| Pro-inflammatory biomarkers | IFN-γ, TNF-α, IL-6, IL-17, IL-1, IL-10, TGF-β | Laboratory analysis of blood |
| Cellular immune response | NK cells and Tregs: IFN, Th1, Th2, Th17, and CTL | Laboratory analysis of blood |

CTL, cytotoxic T-cells; IFN-γ, interferon-gamma; IL, interleukin; NK, natural killer; TGF-β, transforming growth factor-beta; TNF-α, tumor necrosis factor-alpha; Tregs, regulatory T-cells

**SUPPLEMENTARY TABLES**

**Supplementary Table 1** Cytometry antibodies used in the molecular analysis

| Marker | Fluorochrome/color | mAb clone | Panels | Company |
| --- | --- | --- | --- | --- |
| Fc receptor blocker | - | Human Trustain FcX | 1, 2, 3 | BioLegend |
| Fixable viability dye | eFluorUV455 | - | 1, 2, 3 | Invitrogen |
| CD3 | Pacific Blue | HIT3a | 1 | BioLegend |
| CD3 | Alexa Fluor 647 | SK7 | 2 | BioLegend |
| CD4 | APC-Cy7 | OKT4 | 1, 2 | BioLegend |
| CD8 | Brilliant Violet 570 | RPA-T8 | 1, 2 | BioLegend |
| CD56 | Brilliant Violet 711 | 5.1H11 | 1, 2 | BioLegend |
| PD-1 | Brilliant Violet 785 | EH12.2H7 | 1 | BioLegend |
| Tim-3 | PE | F38-2E2 | 1 | BioLegend |
| LAG-3 | PerCP-Cy5.5 | 11C3C65 | 1 | BioLegend |
| CD16 | FITC | 3G8 | 1 | BioLegend |
| CD16 | Pacific Blue | 3G8 | 2 | BioLegend |
| CD25 | PE-Cy7 | BC96 | 1 | BioLegend |
| Foxp3* | Alexa Fluor 647 | 206D | 1 | BioLegend |
| CD44 | Alexa Fluor 700 | IM7 | 1 | BioLegend |
| CD107a | BV785 | H4A3 | 2 | BioLegend |
| IL-17* | PE | BL168 | 2 | BioLegend |
| IFN-g* | PE-Cy7 | 4S.B3 | 2 | BioLegend |
| IL-4* | PerCP-Cy5.5 | MP4-25D2 | 2 | BioLegend |
| Granzyme B* | Alexa Fluor 700 | QA16A02 | 2 | BioLegend |
| Perforin* | FITC | dG9 | 2 | BioLegend |
| CD11b | PE-Cy7 | ICRF44 | 3 | BioLegend |
| CD64 | AF700 | 10.1 | 3 | BioLegend |
| CD80 | BD-V450/P.B. | L307.4 | 3 | BD |
| CD163 | FITC | GHI/61 | 3 | BioLegend |
| CD23 | PerCp-Cy5.5 | EBVCS-5 | 3 | BioLegend |
| CD15 | PE | HI98 | 3 | BioLegend |
| CD33 | APC | WM53 | 3 | BioLegend |
| CD14 | APC/Cy7 | HCD14 | 3 | BioLegend |
| HLA-DR | BV785 | L243 | 3 | BioLegend |
| CD68* | BV711 | Y1/82A | 3 | BD |

The Fc blocker, fixable viability dye, and individual monoclonal antibody used for each panel are shown. Panel 1, T-cell subsets and natural killer cells plus their cell surface-expressing activation or exhaustion markers. Panel 2, T-cell subsets and natural killer cells plus expression of degranulating marker (CD107a) and intracellular cytokines. Panel 3, myeloid plus myeloid-derived suppressor cell populations. *Intracellular markers.

**Supplementary Table 2** Patient demographics and clinical characteristics at baseline

|  | Control group | Yoga group | *p*-value |
| --- | --- | --- | --- |
| Sample size, n | 14 (54%) | 12 (46%) |  |
| Age, years | 60 (59–61) | 56 (55–60.5) | 0.64 |
| Body mass index | 29.4 (37–29.8) | 31.9 (29.5–36.2) | <0.001 |
| T stage |  |  |  |
| 1 | 1 (8 %) | 1 (10%) |  |
| 2 | 6 (50%) | 4 (40%) |  |
| 3 | 5 (42%) | 5 (50%) | 0.92 |
| N stage |  |  |  |
| 0 | 9 (75%) | 10 (100%) |  |
| 1 | 1 (8%) | 0 (0%) |  |
| X | 2 (17%) | 0 (0%) | 0.65 |
| M stage |  |  |  |
| 0 | 4 (33%) | 4 (40%) |  |
| X | 8 (67%) | 6 (60%) | 0.75 |
| Race |  |  |  |
| Asian | 0 (0%) | 1 (8%) |  |
| Black | 0 (0%) | 5 (42%) |  |
| White | 14 (100%) | 6 (50%) | 0.05 |
| Ethnicity |  |  |  |
| Hispanic | 3 (21%) | 3 (25%) |  |
| Non-Hispanic | 11 (79%) | 9 (75%) | 0.83 |
| Postoperative complications |  |  |  |
| Yes | 0 (0%) | 1 (11%) |  |
| No | 11 (100%) | 8 (89%) | 0.26 |

**Supplementary Table 3** Patient-reported outcomes by study group at the scale, sub-scale, and domain levels

|  |  |  | |  |  |
| --- | --- | --- | --- | --- | --- |
| Scale | Domain | Yoga Group  Mean (SE) | Control Group  Mean (SE) | *P*-value | MID |
| Expanded Prostate Cancer Index Composite | |  |  |  |  |
|  | Sexual | 52.1 (7.4) | 56.8 (6.8) | 0.642 | 8.2 |
|  | Urinary | 86.9 (3.8) | 87.0 (2.3) | 0.990 | 3.6 |
| Functional Assessment of Chronic Illness Therapy | |  |  |  |  |
|  | Trial Outcome Index | 82.4 (3.9) | 76.3 (5.2) | 0.359 | 5.5 |
|  | Fatigue | 80.6 (3.4) | 76.4 (4.6) | 0.471 | 4.8 |
| Functional Assessment of Cancer Therapy | |  |  |  |  |
|  | Trial Outcome Index | 80.8 (4.4) | 76.0 (4.3) | 0.444 | 5.0 |
|  | General | 80.0 (3.5) | 78.7 (4) | 0.807 | 4.4 |
|  | Prostate | 79.4 (3.8) | 76.2 (3.9) | 0.563 | 4.5 |
| Five Facet Mindfulness Questionnaire | | 72.9 (3.5) | 68.1 (3.5) | 0.341 | 4.1 |
|  |  |  |  |  |  |

| Sub-Scale | Domain |  | |  |  |
| --- | --- | --- | --- | --- | --- |
| Expanded Prostate Cancer Index Composite | |  |  |  |  |
|  | Sexual bother | 57.3 (10.0) | 62.5 (8.9) | 0.701 | 10.9 |
|  | Sexual function | 49.8 (6.5) | 54.3 (6.3) | 0.626 | 7.5 |
|  | Urinary bother | 82.1 (4.5) | 82.4 (2.9) | 0.960 | 4.3 |
|  | Urinary function | 93.6 (3.3) | 93.3 (2.5) | 0.954 | 3.3 |
|  | Urinary incontinence | 92.0 (4.2) | 97.4 (2.6) | 0.279 | 4.0 |
|  | Urinary obstructive | 85.4 (3.5) | 82.1 (3.2) | 0.500 | 3.9 |
| Functional Assessment of Cancer Therapy | |  |  |  |  |
|  | Emotional wellbeing | 74.7 (4.3) | 70.2 (4.6) | 0.487 | 5.2 |
|  | Fatigue | 81.7 (4.0) | 71.6 (6.5) | 0.196 | 6.5 |
|  | Functional wellbeing | 80.1 (4.9) | 74.5 (6.2) | 0.488 | 6.6 |
|  | Prostate Cancer Subscale | 78.2 (5.0) | 70.7 (4.7) | 0.285 | 5.7 |
|  | Physical wellbeing | 86.0 (3.9) | 86.8 (3.9) | 0.885 | 4.5 |
|  | Social wellbeing | 78.6 (4.5) | 82.1 (3.9) | 0.556 | 4.9 |
| Five Facet Mindfulness Questionnaire | |  |  |  |  |
|  | Awareness | 77.3 (3.3) | 70.7 (5.1) | 0.281 | 5.1 |
|  | Describing | 77.6 (5.9) | 71.4 (6.2) | 0.476 | 7.1 |
|  | Nonjudging | 80.2 (4.5) | 74.5 (5.4) | 0.430 | 5.9 |
|  | Nonreactive | 66.7 (4.1) | 61.0 (3.5) | 0.303 | 4.5 |
|  | Observing | 62.0 (4.7) | 62.0 (4.2) | 0.995 | 5.2 |

**Supplementary Table 4**. Comparison of changes from baseline in levels of 38 cytokines between the yoga group and the control group.

| Cytokine Name | Standard Arm | Yoga Arm | Effect Size with 95% Confidence Intervals | P-values |
| --- | --- | --- | --- | --- |
| EGF | 0.02 | -0.22 | -0.23 (-1.08, 0.62) | 0.549 |
| FGF2 | -0.2 | -0.49 | -0.28 (-1.29, 0.72) | 0.549 |
| Eotaxin | 0.02 | -0.02 | -0.03 (-0.34, 0.27) | 0.815 |
| TGF-a | -0.07 | -0.05 | 0.02 (-0.17, 0.21) | 0.829 |
| G-CSF | 0.14 | -0.41 | -0.55 (-1.05, -0.05) | 0.032 |
| FLt-3L | 0.67 | -0.23 | -0.91 (-1.83, 0.01) | 0.053 |
| GM-CSF | 0.03 | -0.15 | -0.18 (-0.66, 0.31) | 0.446 |
| Fractalkine | -0.54 | -0.43 | 0.11 (-1.27, 1.5) | 0.867 |
| IFNa2 | 0.04 | -0.11 | -0.14 (-0.5, 0.22) | 0.415 |
| IFNy | -0.13 | -0.28 | -0.15 (-0.59, 0.29) | 0.48 |
| GRO | 0.67 | 0.60 | -0.07 (-1.57, 1.43) | 0.923 |
| IL-10 | 0.08 | -0.07 | -0.15 (-0.53, 0.23) | 0.392 |
| MCP-3 | -0.05 | -0.05 | 0 (-0.19, 0.19) | 0.988 |
| IL-12 P40 | 0.03 | -0.02 | -0.05 (-0.37, 0.27) | 0.763 |
| MDC | 0.10 | 0.05 | -0.05 (-0.3, 0.2) | 0.667 |
| IL-12P70 | -0.23 | 0.07 | 0.3 (-0.26, 0.86) | 0.27 |
| IL-13 | -0.06 | -0.10 | -0.04 (-0.39, 0.3) | 0.793 |
| IL-15 | 0.12 | 0.06 | -0.07 (-0.35, 0.21) | 0.612 |
| sCD40L | 0.21 | 0.23 | 0.02 (-0.54, 0.58) | 0.938 |
| IL-17A | -0.14 | 0.08 | 0.22 (-0.14, 0.58) | 0.212 |
| IL-1RA | 0.15 | 0.02 | -0.13 (-0.69, 0.43) | 0.632 |
| IL-1A | -0.15 | -0.42 | -0.28 (-1, 0.45) | 0.422 |
| IL-9 | -0.02 | -0.09 | -0.08 (-0.38, 0.23) | 0.608 |
| IL-1B | -0.07 | 0.00 | 0.07 (-0.04, 0.18) | 0.197 |
| IL-2 | -0.05 | -0.03 | 0.02 (-0.11, 0.15) | 0.733 |
| IL-3 | -0.01 | -0.02 | -0.01 (-0.23, 0.21) | 0.931 |
| IL-4 | 0.09 | -0.12 | -0.21 (-0.59, 0.18) | 0.276 |
| IL-5 | 0.02 | -0.03 | -0.05 (-0.33, 0.23) | 0.723 |
| IL-6 | -0.11 | -0.15 | -0.05 (-0.28, 0.18) | 0.66 |
| IL-7 | -0.05 | -0.09 | -0.04 (-0.28, 0.2) | 0.736 |
| IL-8 | 0 | -0.28 | -0.28 (-0.73, 0.16) | 0.195 |
| IP-10 | -0.02 | -0.22 | -0.2 (-0.59, 0.18) | 0.278 |
| MCP-1 | 0.1 | -0.12 | -0.22 (-0.44, -0.01) | 0.044 |
| MIP-1a | 0.09 | -0.13 | -0.23 (-0.75, 0.3) | 0.374 |
| MIP-1B | -0.09 | -0.42 | -0.33 (-1.07, 0.4) | 0.336 |
| TNFa | 0.03 | -0.01 | -0.03 (-0.21, 0.14) | 0.694 |
| TNFb | -0.01 | -0.12 | -0.12 (-0.49, 0.26) | 0.526 |
| VEGF | -0.23 | -0.05 | 0.18 (-0.92, 1.28) | 0.722 |

**
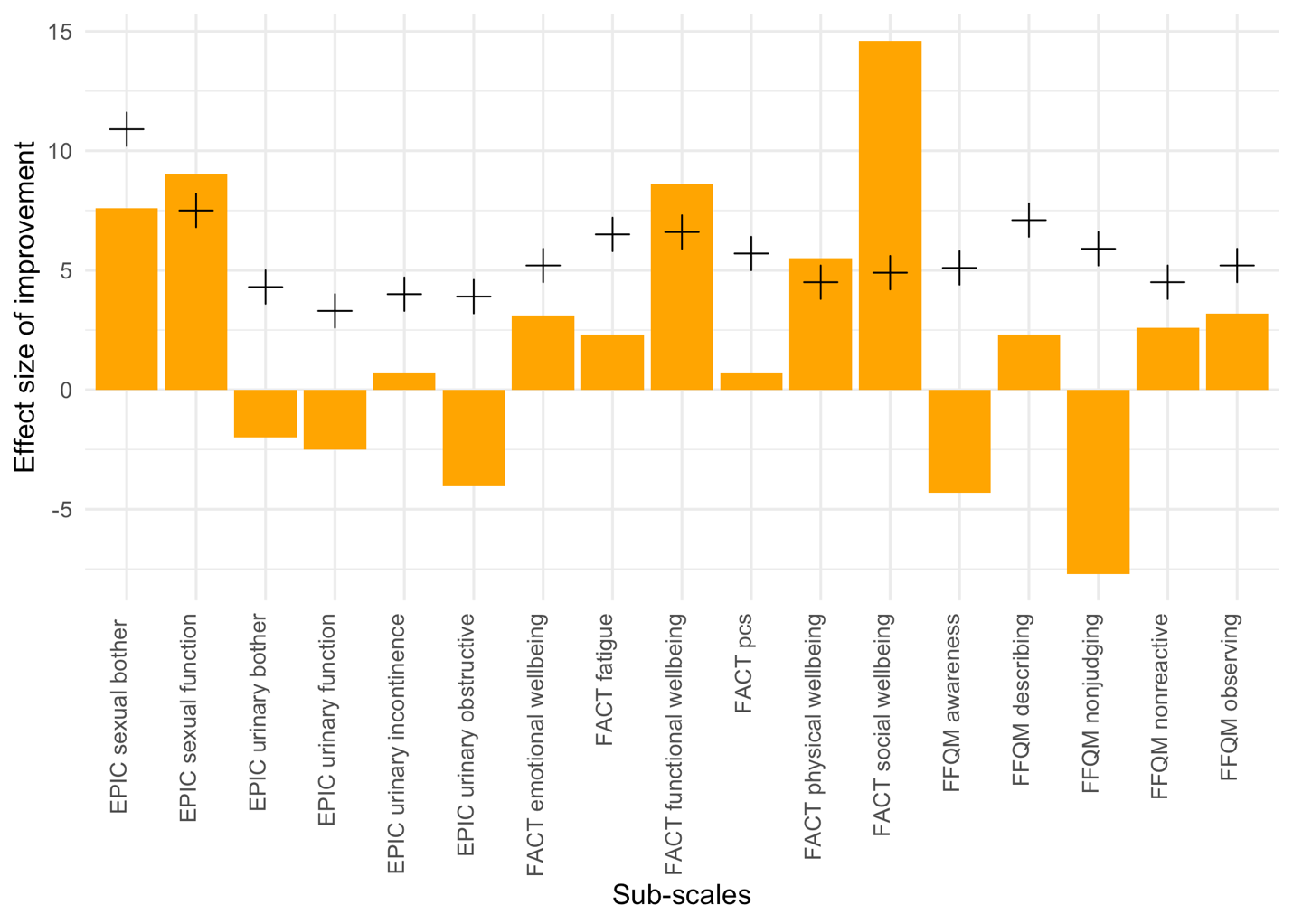
**

**Supplementary Figure 1.** Effect size of the yoga intervention versus the minimally important difference. *The effect size was calculated as the difference between the post-intervention score minus the pre-intervention score in the yoga group and the post-intervention score minus the pre-intervention score in the control group. The MID was calculated as one-third of the overall standard deviation of the patient-reported scale item at baseline. The ‘+’ mark on the graph indicates the MID for the subscale item. EPIC, Expanded Prostate Index Composite; FACIT, Functional Assessment of Chronic Illness Therapy; FACIT-F, FACIT-Fatigue; FACIT-G, FACIT-General; FACT, Functional Assessment of Chronic Illness Therapy; FFOM, Five Facets of Mindfulness; MID, minimally important difference

**(a)**

**
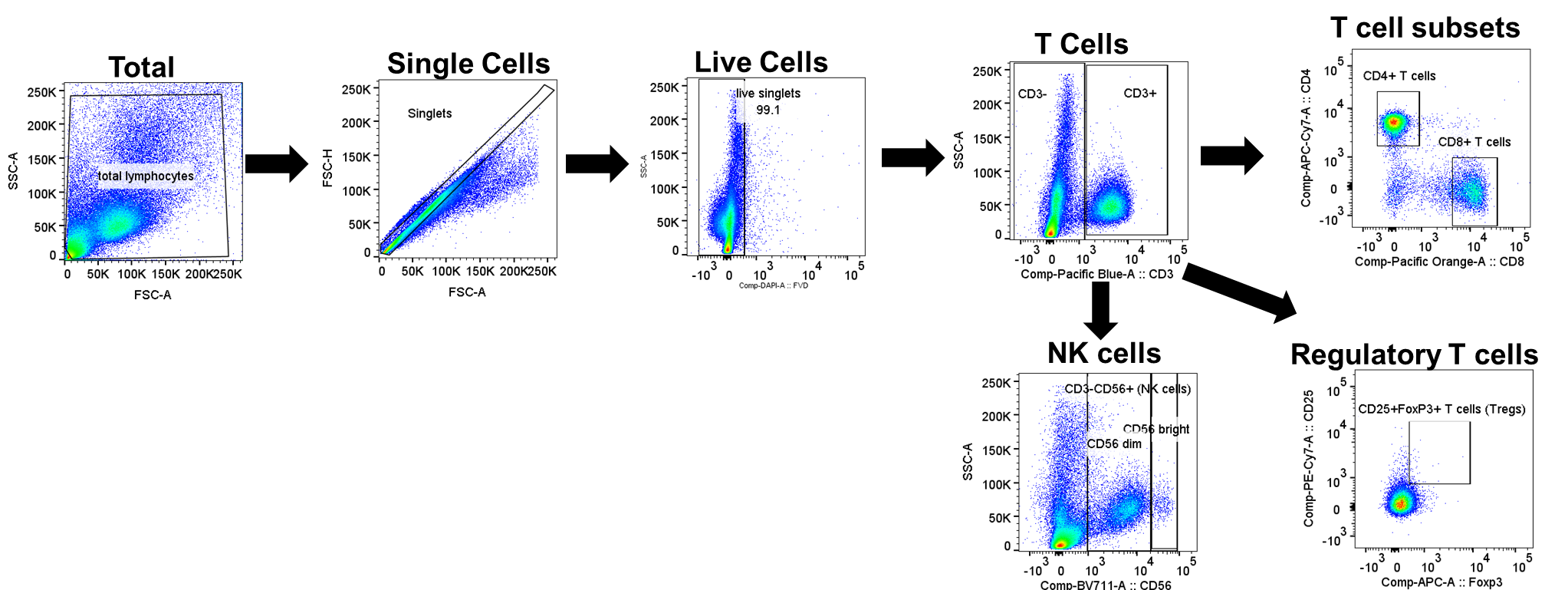
**

**(b)**

**
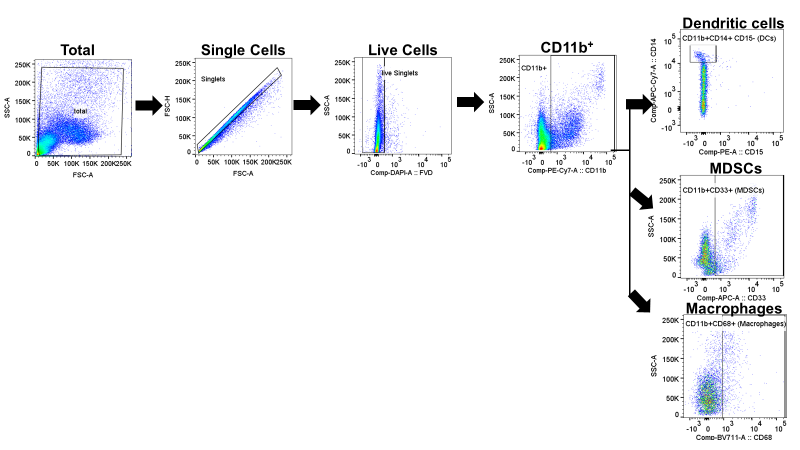
**

**(c)**

**
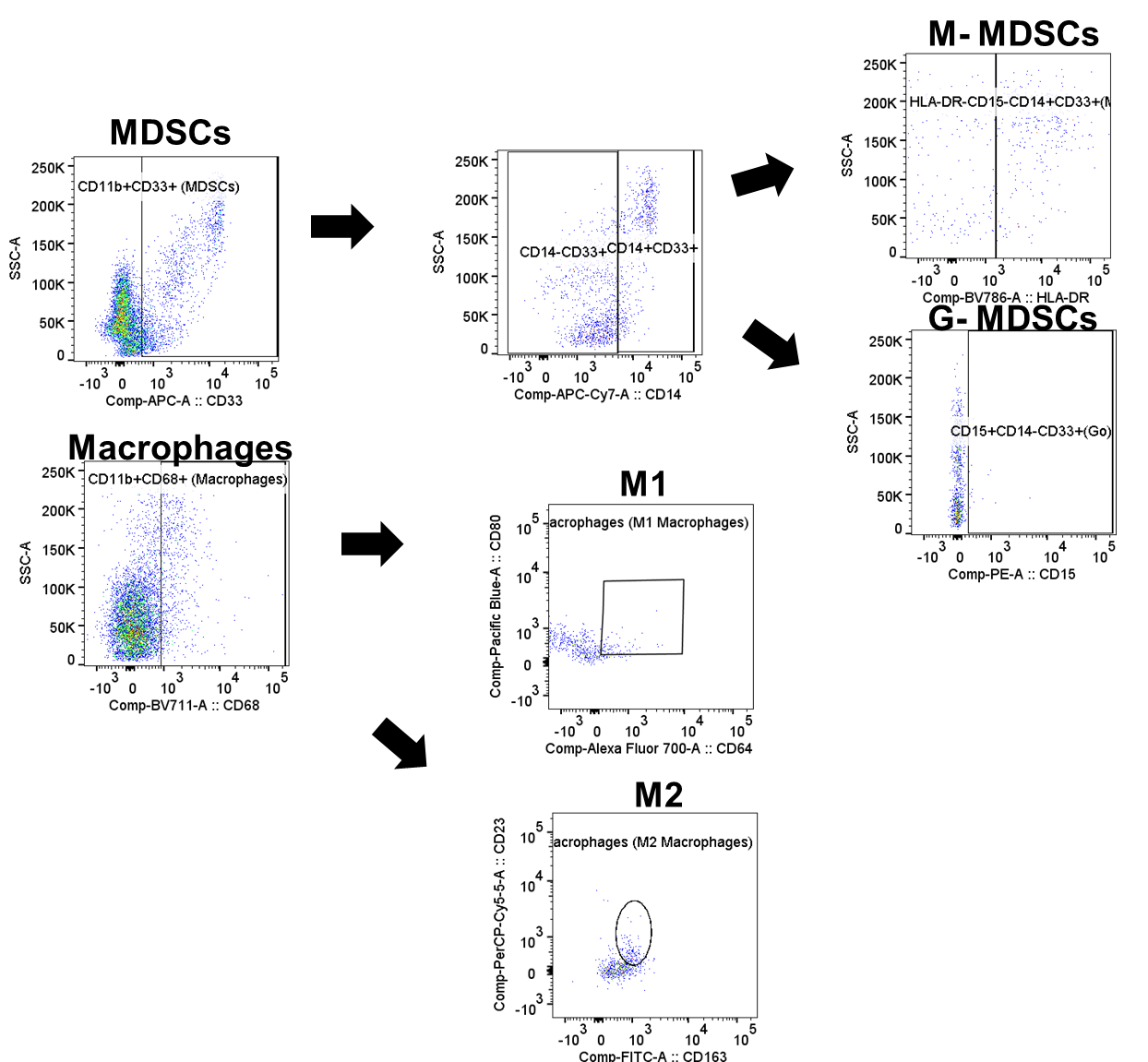
**

**Supplementary Figure 2.** Characterization of lymphocytes in blood samples using multi-parametric gating flow cytometry. (**a**) Gating strategy for T-cells and NK cells. Dot plots showing the gating strategy as used to identify myeloid cell populations in PBMCs as a representative example. First, doublets were eliminated using a pulse geometry gate (FSC-A vs. FSC-H). Next, live or dead cells were gated using fixable viability dye. T-cells were identified as live CD3+ cells and CD4+, CD8+, or regulatory T-cells (Tregs) as CD25+FOXP3+ T-cells. NK cells were identified as CD3-CD56+ cells and further divided into CD56^bright^ and CD56^dim^ NK cells. (**b**) Gating strategy for subsets of myeloid cells. Dot plots showing the selected gating strategy used to identify the myeloid cell populations in PBMCs as a representative example. First, doublets were eliminated using a pulse geometry gate (FSC-A vs. FSC-H). Next, live or dead cells were using fixable viability dye. Dendritic cells were identified as CD11b+CD14+CD15- cells, myeloid-derived suppressor cells (MDSCs) as CD11b+CD33+ cells and macrophages as CD11b+CD68+ cells. (**c**) Gating strategy of myeloid-derived suppressor cell subsets and macrophage subsets. Myeloid-derived suppressor cells (CD45+CD11b+CD33+ cells) were divided into M-MDSCs, identified as HLADR-CD15-CD14+CD33+CD11b+ cells, and G-MDSCs, identified as CD15+CD14-CD33+CD11b+ cells. Macrophages (CD11b+CD68+ cells) were divided into M1 macrophages (CD64+CD80+CD11b+CD68+ cells) and M2 macrophages (CD163+CD23+CD11b+CD68+ cells). MDSCs, myeloid-derived suppressor cells; NK cells, natural killer cells; PBMCs, peripheral blood mononuclear cells


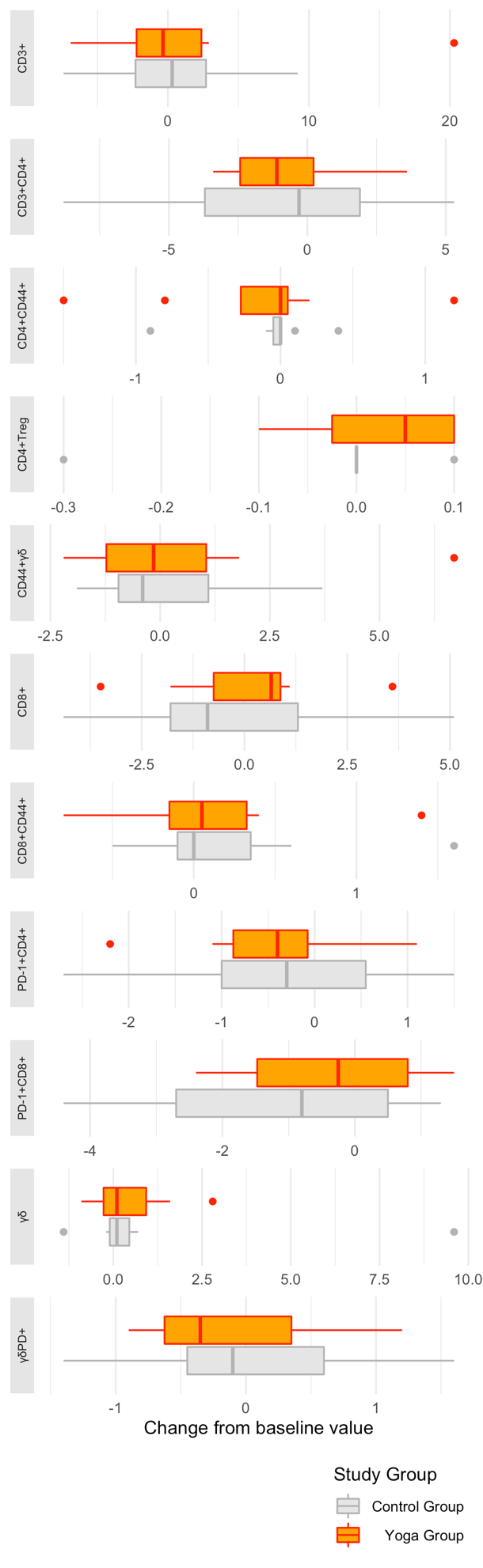
(**a**)


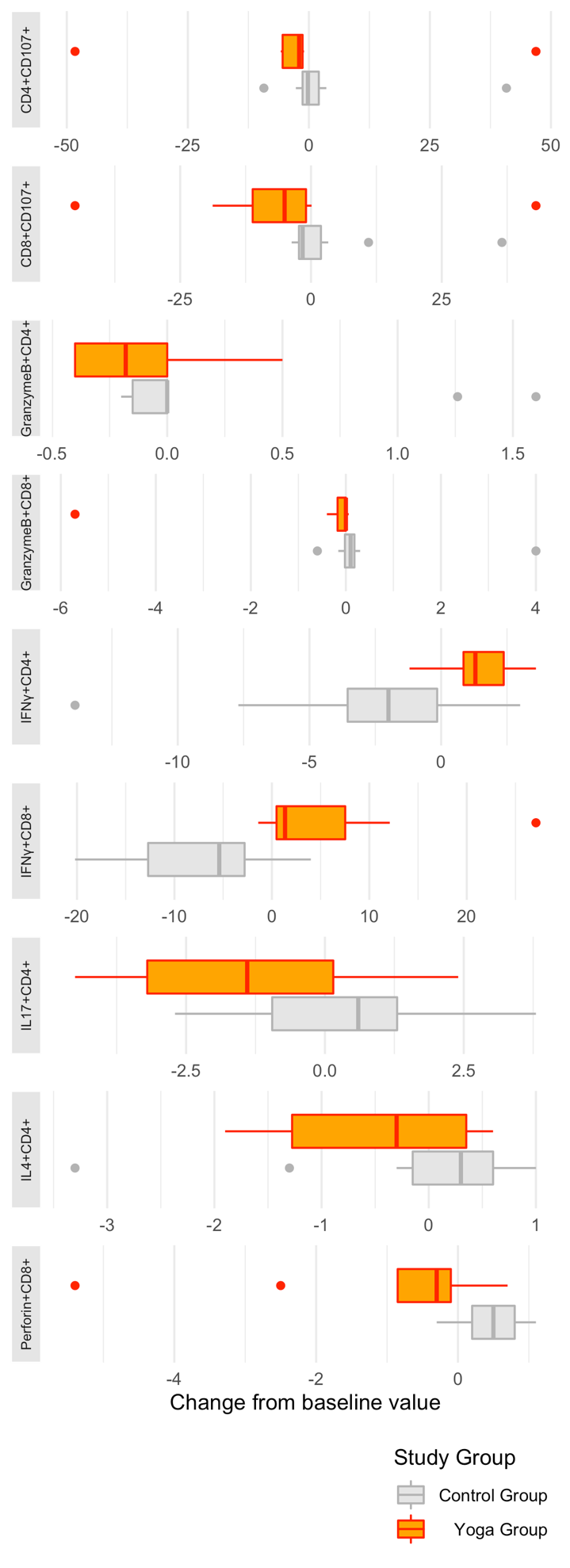
(**b**)


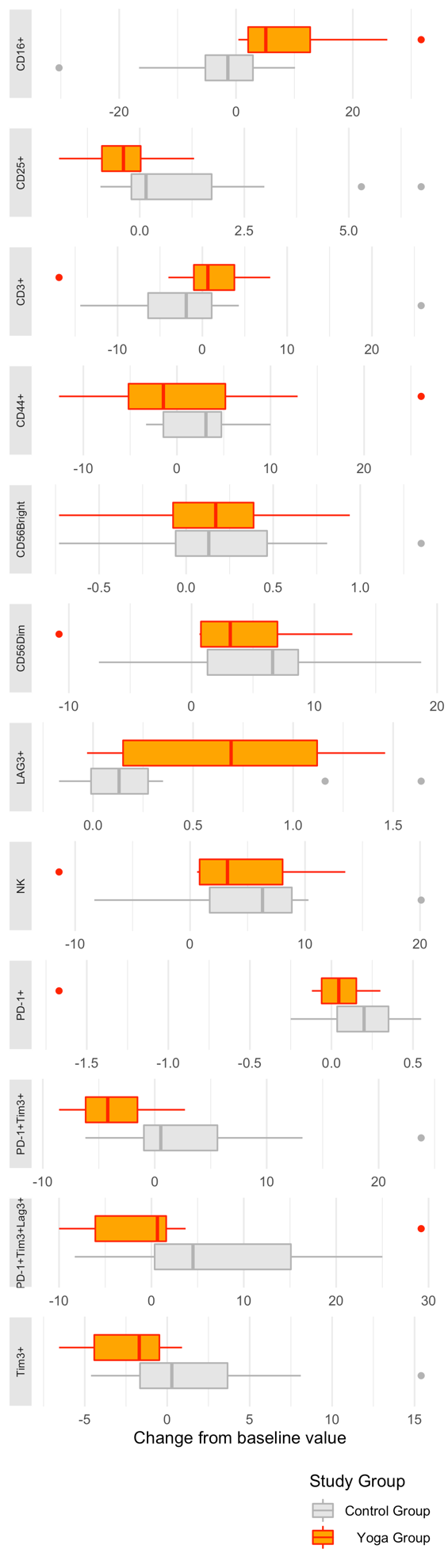
(**c**)


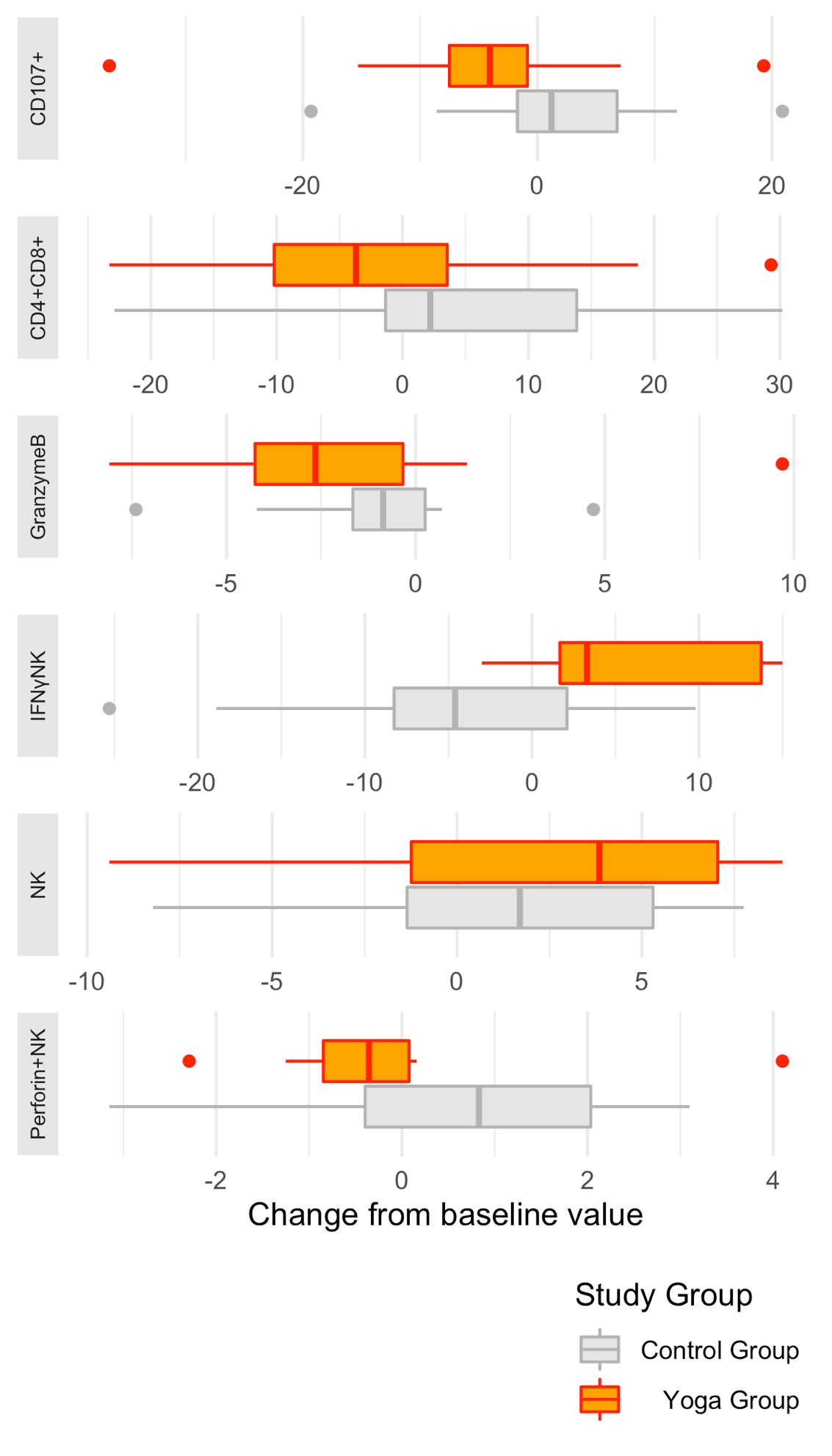
(**d**)


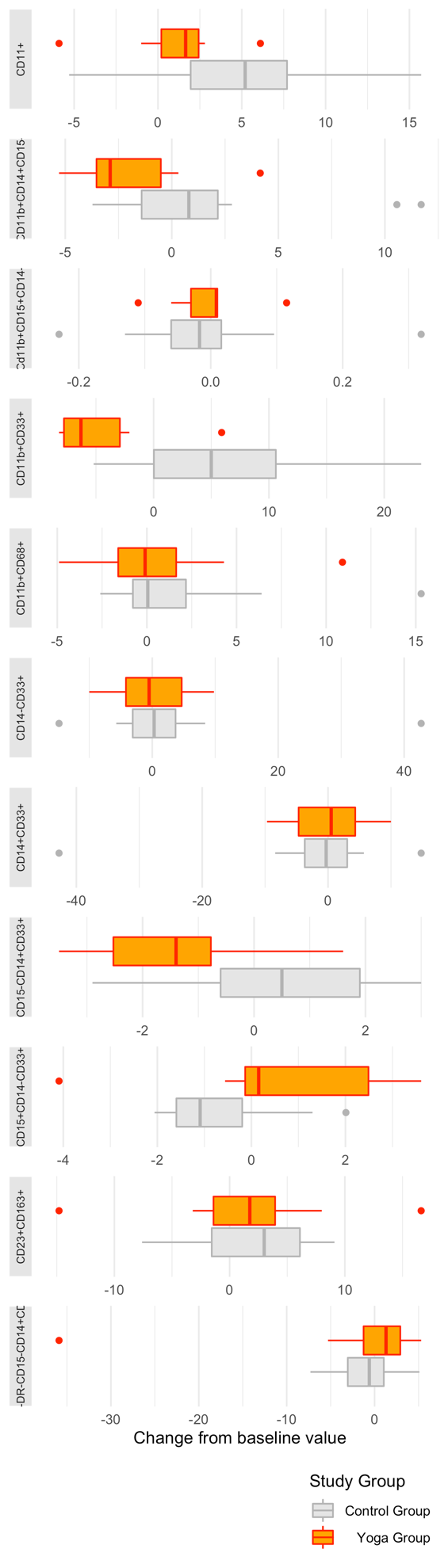
(**e**)

**Supplementary Figure 3.** Box and whisker plots comparing the changes in frequency and absolute numbers of immune cells between the yoga group and the control group.

(**a, b**) T-cell data showing an increased IFN-γ response in peripheral cytotoxic CD4+ and CD8+ cells and decreased levels of regulatory T-cells, indicating better antitumor activity in the yoga group. (**c, d**) NK cell data showing increased Fc receptor III and IFN-γ expression in the yoga group, indicating a robust immune response. (**e**) Myeloid cell data showing a decrease in MDSCs, also indicating increased antitumor activity in the yoga group. IFN-γ, interferon-gamma; MDSCs, myeloid-derived suppressor cells; NK, natural killer


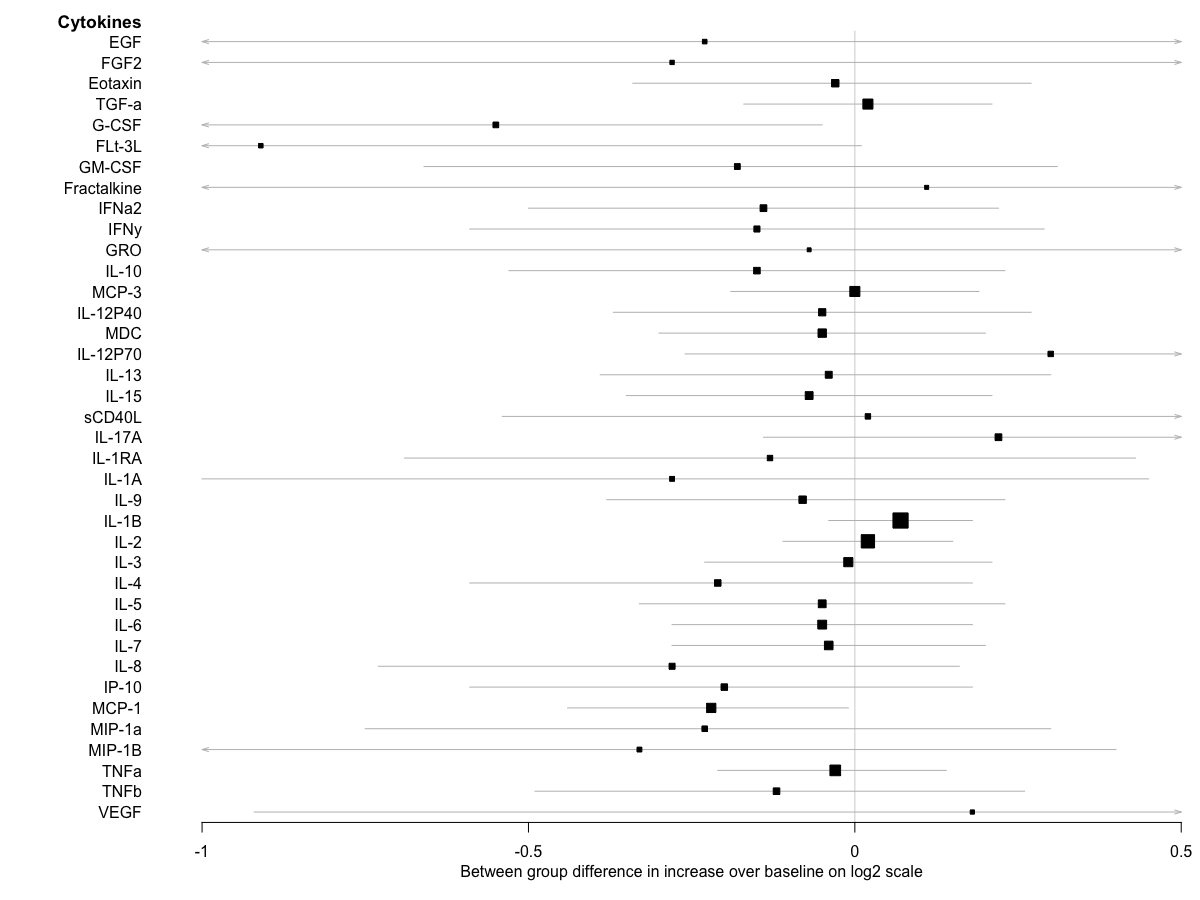


**Supplementary Figure 4**. Comparison of changes from baseline in levels of 38 cytokines between the yoga group and the control group.
